# Hormonal and Thirst Modulated Maintenance of Fluid Balance in Young Women with Different Levels of Habitual Fluid Consumption

**DOI:** 10.3390/nu8050302

**Published:** 2016-05-18

**Authors:** Evan C. Johnson, Colleen X. Muñoz, Liliana Jimenez, Laurent Le Bellego, Brian R. Kupchak, William J. Kraemer, Douglas J. Casa, Carl M. Maresh, Lawrence E. Armstrong

**Affiliations:** 1 Human Performance Laboratory, Department of Kinesiology, University of Connecticut, Storrs, CT 06269, USA; cmunoz@hartford.edu (C.X.M.); Brian.kupchak@gmail.com (B.R.K.); kraemer.44@osu.edu (W.J.K.); douglas.casa@uconn.edu (D.J.C.); maresh.15@osu.edu (C.M.M.); lawrence.armstrong@uconn.edu (L.E.A.); 2 Division of Kinesiology and Health, University of Wyoming, Laramie, WY 82071, USA; 3 Department of Health Sciences and Nursing, University of Hartford, West Hartford, CT 06117, USA; 4 Hydration & Health Department, Danone Research, Palaiseau 91767, France; liliana.jimenez@danone.com (L.J.); laurent.le-bellego@danone.com (L.L.B.); 5 Department of Military and Emergency Medicine, Uniformed Services University of the Health Sciences, Bethesda, MD 20814, USA; 6 Department of Human Sciences, the Ohio State University, Columbus, OH 43210, USA

**Keywords:** arginine vasopressin, aldosterone, thirst, osmolality, urine

## Abstract

Background: Surprisingly little is known about the physiological and perceptual differences of women who consume different volumes of water each day. The purposes of this investigation were to (a) analyze blood osmolality, arginine vasopressin (AVP), and aldosterone; (b) assess the responses of physiological, thirst, and hydration indices; and (c) compare the responses of individuals with high and low total water intake (TWI; HIGH and LOW, respectively) when consuming similar volumes of water each day and when their habitual total water intake was modified. Methods: In a single-blind controlled experiment, we measured the 24 h total water intake (TWI; water + beverages + food moisture) of 120 young women. Those who consumed the highest (HIGH, 3.2 ± 0.6 L·day^−1^, mean ± SD) and the lowest (LOW, 1.6 ± 0.5 L·day^−1^) mean habitual TWI were identified and compared. Outcome variables were measured during two *ad libitum* baseline days, a four-day intervention of either decreased TWI (HIGH) or increased TWI (LOW), and one *ad libitum* recovery day. Results: During the four-day intervention, HIGH and LOW experienced differences in thirst (*p* = 0.002); also, a statistically significant change of AVP occurred (main effect of TWI and day, *p* < 0.001), with no effect (TWI or day) on aldosterone and serum osmolality. Urine osmolality and volume distinguished HIGH from LOW (*p* = 0.002) when they consumed similar 24 h TWI.

## 1. Introduction

Homeostasis of human body fluid is achieved through behavioral and hormonal negative feedback and feed-forward mechanisms which regulate body fluid osmolality, blood volume, blood plasma sodium concentration, and blood pressure in response to alterations in either intra- or extracellular water volume [1,2]. The most prominent effectors of body water balance are the perception of thirst, assuming the subsequent consumption of water, and the circulating concentration of hormones such as arginine vasopressin (AVP) and those related to the renin angiotensin aldosterone system (RAAS; *i.e.*, angiotensin II, aldosterone, *etc*.) [3]. The directionality of any disturbance to the above variables determines if the individual behaviorally seeks out water while physiologically conserving body water through excretion of small volumes of concentrated urine, or if the individual abstains from water intake while freely excreting waste along with larger volumes of dilute urine. The coordination of these mechanisms allows, under normal circumstances, for acutely unperturbed physiological capacity, as evidenced by tight control of blood osmolality, across a wide range of daily fluid intakes influenced by both regulation of body fluid and non-regulatory drinking [4,5]. However, it has begun to be determined that physiological differences exist (*i.e.*, circulating levels of AVP) between the members that habitually consume fluid at the top (high drinkers; HIGH) *versus* the bottom end (low drinkers; LOW) of this continuum [6]; if this is true, adaptations to chronic high internal water availability or low availability could affect how members of the groups respond to changes in water intake.

Humans’ capability to function normally across a wide range of daily fluid intakes has resulted in the scientific community’s ability to suggest daily water intake only as “adequate intake” as opposed to the “recommended intake” associated with less tightly regulated nutrients [5,7]. Thus, many purport that fluid intake above one’s perception of thirst is unnecessary due to the previously mentioned tight control over plasma osmolality [8,9]. In fact, good scientific investigations on body fluid regulation have demonstrated in rodent, dog, and human models that when water is withheld, thirst, plasma AVP, and urine concentration all increase, while urine volume decreases [3,10,11,12]. Conversely, when water loading is practiced by acutely consuming large volumes of water the opposite effect occurs [13,14]. Within these individuals it can be assumed that total body water is conserved *(i.e.*, when little water is consumed, little is excreted and *vice versa*). For this reason, controversy has arisen surrounding scientific findings related to purported benefits of consuming larger volumes of fluid on a daily basis. Although avoidance of low water intake has been linked to such benefits as improved mood [15,16], improved cognition in children [17,18], and reduced risk for hyperglycemia or type 2 diabetes [19,20], the physiological mechanisms behind most of these associations have yet to be detailed [21]. This, combined with mixed findings related to health outcomes and various levels of fluid intake, makes it quite easy to suggest that high drinkers and low drinkers are physiologically similar, but operating (properly so) at opposite ends of the natural body water regulatory system [22,23].

The investigations mentioned above would seem to suggest that there is no difference. However, many previous studies that would support this thought have altered water intake after assuming all participants are equal when consuming their habitual intake. For example, the intricate work of Shore *et al.* randomized participants to three days of either water restriction (1 L∙day^−1^), or to water loading (6.8 L∙day^−1^) followed by three moderate intake days, and then switched treatments. This type of intervention is valuable in terms of characterizing general responses to fluid extremes. However, it does not account for potential differences due to the participants’ previous habitual water intake. Prior research that has accounted for habitual water intake prior to a change in water intake has demonstrated that urinary markers of hydration respond similarly between groups [24,25], however, the drivers of these changes (*i.e.*, hormonal concentrations and perception of thirst) have yet to be characterized. Thus, it is important to further illustrate the perceptual and physiological responses to changes in water intake within the normal physiological range after accounting for previous fluid intake.

The present investigation was designed to clarify differences between free-living healthy women who consume low (LOW, 1.6 ± 0.5 L·day^−1^) and high (HIGH, 3.2 ± 0.6 L·day^−1^) volumes of water during daily activities. The purposes of this research were to (a) analyze blood osmolality, AVP, and aldosterone; (b) assess the perceptual responses of thirst; and (c) compare the responses of HIGH and LOW, when consuming similar volumes of water each day and when their habitual total water intake (TWI) was modified. We hypothesized that during the four-day intervention that AVP concentration (but not serum osmolality or aldosterone) and urinary hydration indices would change significantly in both HIGH and LOW. We also hypothesized that, when consuming a similar 24 h TWI, the LOW and HIGH groups would be similar with regard to all measured variables. If these hypotheses were upheld, then it could be confirmed that the only difference between HIGH and LOW are the concentration and volume of their urine. However, observed differences in response to water intake change would constitute evidence of physiological adaptation to either chronic high or low water intake.

## 2. Experimental Section

120 college-aged women (age, 20 ± 2 years; height, 165 ± 7 cm; body mass, 62.1 ± 11 kg) provided written informed consent to participate. This study was conducted according to the guidelines of the Declaration of Helsinki and all procedures involving human subjects were reviewed and approved by the University of Connecticut Institutional Review Board for human research (Protocol H10-144). The results presented here are a follow-up analysis of an experiment that has resulted in other publications [16,25,26] but includes data, specifically the hormonal data, that has not been presented outside of conference proceedings [27].

### 2.1. Subject Screening and Selection

We identified the habitual daily water intake of 120 healthy young women; all were oral contraceptive users. Participants were instructed to maintain their regular diet over five consecutive days, while recording all food and fluids consumed (days 1–5). Participants returned to the laboratory on days 2–6 between 0530 h and 0800 h to review the previous 24 h diet record with a nutritional consultant. Daily beverage volume (fluid intake, FI) was calculated directly from participant diet records, and 24 h TWI (including moisture in foods) was computed via commercial software (Nutritionist Pro™, Axxya Systems, Stafford, TX, USA).

### 2.2. Study Design

The purpose of the experimental intervention was to compare fluid-electrolyte regulatory hormones and thirst ratings in two distinct subsamples of the original 120 women. The first subsample (HIGH; *n* = 14) habitually consumed the largest 24 h TWI volume (3.2 ± 0.6 L·day^−1^) and the second (LOW; *n* = 14) habitually consumed the smallest daily TWI volume (1.6 ± 0.6 L·day^−1^, one-half of the TWI of HIGH). These women, verified again as oral contraceptive users, provided separate written consent to participate in modified TWI. Selected information was withheld from the participants, including their TWI rank and group status, to avoid influencing or biasing normal fluid consumption during the baseline days.

During the experimental phase of the investigation, women were observed on seven consecutive days of the experimental intervention: two days of baseline *ad libitum* fluid intake (water and other beverages, as preferred; days 1–2), four days of modified fluid intake (controlled, with water as the only beverage permitted on days 3–6), and one recovery day when each group returned to *ad libitum* fluid intake (e.g., consuming water and other beverages as preferred; day 7). During the treatment (days 3–6), the TWI of each group was modified to approximately match the habitual mean TWI of the other group. Although both groups of 14 women were observed during periods of large and small water intake, the group names HIGH and LOW are used in this manuscript to represent habitual baseline TWI.

Participants reported to the laboratory between 05:30 h and 09:00 h. Arrival time did not differ more than 1 h on all experimental days. Day 1 was individualized for each participant, occurring on the self-reported first placebo day of her oral contraceptive pill pack; this allowed testing to occur when plasma estrogen and progesterone concentrations were relatively low. Beginning on the morning of Day 1, subjects collected consecutive 24 h urine samples that were returned during each subsequent morning laboratory visit. On the morning of days 1–8, one blood sample was drawn from an antecubital vein via single venipuncture, and was collected in serum and plasma EDTA tubes (Becton, Dickson and Company, Franklin Lakes, NJ, USA). Filled serum tubes were allowed to clot at room temperature. Next, all blood samples were centrifuged at 5000 rpm for 15 min at −4 °C (Hettich, Rotina 35 R, Tuttlingen, Germany). Serum osmolality was measured immediately following centrifugation. The remaining serum and all plasma samples were separated into aliquots and frozen at −80 °C until additional assays were performed within three months, as described below. All 24 h urine collections (days 1–7) were analyzed for total volume by mass (Ohaus, Ranger, Parsippany, NJ, USA), and urine osmolality by freezing point depression (Advanced Instruments Inc., Model 3320, Norwood, MA, USA).

Some variables were measured across seven continuous 24 h periods, and others were evaluated on eight consecutive mornings. Specifically, TWI and urine collections were evaluated from one morning to the next, during seven 24 h periods (*i.e.*, days 1–2, 2–3, 3–4, 4–5, 5–6, 6–7, and 7–8). Body mass (±100 g; Health O Meter^®^, Model 349KLX, Alsip, IL, USA), blood variables, and perceived thirst were recorded during each of eight morning visits, on days 1–8. Thus, three baseline measures were obtained on these acute variables (body mass, blood variables, and thirst) because the third measurement occurred on the morning of day 3, immediately prior to the start of modified fluid intake. All variables were finalized on the morning of day 8 because they involved response to the recovery day (day 7). Thirst was assessed with a 9-point Likert scale [28] which included five written descriptors of thirst and required that subjects select one number, from 1 to 9.

During the intervention (days 3–6), participants were instructed to maintain normal solid food choices, but to consume water as their only fluid. Members of HIGH were instructed to reduce their fluid intake to 1.3 L·day^−1^, and LOW were instructed to increase their fluid intake to 3.0 L·day^−1^; this was accomplished by drinking only mineral water (Volvic^®^ mineral water, Danone, France) that was provided each morning in plastic bottles. This water contained a low mineral content (mg·L^−1^; sodium, 9; chloride, 8; potassium, 6; sulphates, 9; magnesium, 6; nitrate, 1; silica, 30). Due to additional moisture in solid foods, the TWI per 24 h was greater than these volumes.

Plasma arginine vasopressin (AVP) was analyzed by enzyme immunoassay (Cayman Chemical, Ann Arbour, MI, USA), with sensitivity of 23.0 pg·mL^−1^. Purification and concentration of samples twenty-fold, in accordance with the manufacturer’s instructions, was performed to bring the AVP concentrations into a detectable range. The intra-assay coefficient of variation (CV) was 7.4% and the inter-assay CV was 8.1%. The assay wavelength was read at 405 nm on a tunable microplate reader (Molecular Devices, VERSAmax, Sunnyvale, CA, USA). Serum aldosterone was analyzed by Enzyme-linked immunosorbent assay (ALPCO, Salem, NH, USA) in accordance with the manufacturer’s instructions, with a sensitivity of 15.0 pg·mL^−1^, an intra-assay CV of 9.2%, and inter-assay CV of 6.2%. The assay wavelength was read at 450 nm on the microplate reader described above.

### 2.3. Statistical Analysis

The initial sample size of 120 women was chosen in an effort to achieve a sample mean similar to that of previous investigations [29] and to allow for sufficient members of HIGH and LOW. The initial sample size was calculated using the equation *n* = (*Z*_α/2_·σ/*E*)^2^ where *z* = critical value (1.96), σ = expected standard deviation (1.03 L) based on the above reference, and *E* = the allowed margin of error (0.20 L). Given this equation, the calculated minimum sample size was 102. For the intervention, a critical sample size of four participants was calculated, based on previous observations of AVP during water deprivation [30], with μ_1_ = 0.5 pmol·L^−1^, μ_2_ = 3.0 pmol·L^−1^, σ = 1.0, and β = 0.9.

Commercially-available software (SPSS Statistics 17.0, SPSS, Inc., Chicago, IL, USA) was utilized to perform all statistical analyses. Two-way (TWI × group) repeated measure analysis of variance (ANOVA) was used to test each dependent variable during the fluid modification period. In this instance, TWI refers to the fluid volume that either HIGH or LOW consumed during the treatment. For example, HIGH consumed a larger fluid volume during days 1–2, but LOW consumed a larger fluid volume during days 3–6. Therefore, the first time point entered in the statistical analysis represented the mean values from days 1–2 for HIGH, and days 3–6 for LOW. Accordingly, the second time point corresponded to the mean value of days when both groups were consuming a smaller TWI (HIGH, days 3–6; LOW, days 1–2). Days 7–8 were excluded from this analysis. Sphericity was assumed for within-subject effects unless Mauchley’s test of sphericity was violated, in which case the Greenhouse-Geisser correction was used. When an effect of group was noted with ANOVA, *post-hoc* independent sample *t*-tests were used to compare HIGH and LOW groups at specific TWI. When main effects of fluid volume or an interaction effect (TWI × group) was noted, *post-hoc* paired-sample *t*-tests were used to compare days 1–2 *versus* days 3–6 within HIGH and LOW.

The volume of fluid prescribed to HIGH on days 3–6 resulted in a higher TWI than the volume consumed by LOW on days 1–2. Therefore, dependent variable ANOVAs that would be directly influenced by this discrepancy (urine volume, urine osmolality) were corrected using the absolute value of TWI change (*i.e.*, days 1–2 *versus* days 3–6) as a covariate during analyses of covariance (ANCOVA).

Fluid regulatory hormone concentrations were analyzed separately for HIGH and LOW via one way repeated measures ANOVA across eight time points: three during *ad libitum* baseline (morning mean values of days 1–3), four during the treatment (morning mean values of days 4–7), and one during recovery (day 8) to describe the time course of responses to abrupt changes in TWI. When a main effect of time was detected, simple contrasts were used to compare individual data points to the baseline mean.

Lastly, the relationship between AVP and perception of thirst values was compared between groups via individual regression analysis. Each participant provided two values to the analysis, a mean value during small fluid intake and a mean value during large fluid intake. The individual lines of regression were then compared for similarities of slope and y-intercept [31].

## 3. Results

### 3.1. Full Sample Fluid Intake

**Figure 1:**
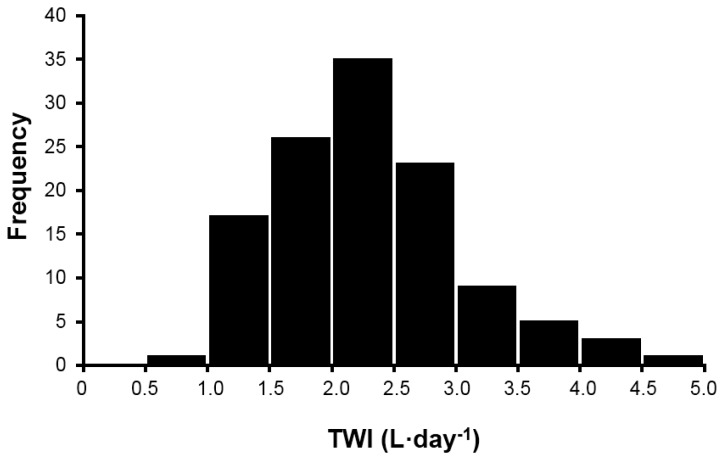
Frequency distribution of TWI (mean of five days) for 120 healthy, young college-aged women.

Measurements across five days allowed investigators to determine the TWI of 120 healthy young women, with the intent to compare these findings to published recommendations of daily adequate intake of water for women [5,7]. Figure 1 presents the 24 h TWI (L·day^−1^) frequency distribution (mean of five days) of the original sample of 120 women, during screening. Although this distribution (mean ± SD for TWI was 2.3 ± 0.8 L·day^−1^) is moderately, positively skewed (skewness score, 0.62) and is slightly leptokurtic (excess kurtosis 0.33), neither characteristic influenced the selection of test participants. Based on *ad libitum* TWI, the 14 women in HIGH (age, 21 ± 2 years; height 166 ± 10 cm; mass, 65.1 ± 13.0 kg) were selected because they habitually consumed a large 24 h TWI volume (3.2 ± 0.6 L·day^−1^). The 14 women in LOW (age, 20 ± 1 years; height; 162 ± 8 cm, mass, 55.3 ± 7.2 kg) were selected because they habitually consumed a small daily TWI volume (1.6 ± 0.4 L·day^−1^, one-half of the TWI of HIGH).

### 3.2. Treatment Fluid Intake

Fluid volumes consumed by HIGH and LOW can be found in (Table 1). HIGH consumed 63% of baseline TWI (2.0 ± 0.2 L·day^−1^), and LOW consumed 219% more than baseline TWI (3.5 ± 0.1 L·day^−1^). Baseline TWI volumes did not differ from those observed during the screening period. A main effect of TWI (L·day^−1^) was observed as HIGH and LOW modified their daily TWI (*F*(1, 26) = 33.08, *p* < 0.001). A significant interaction effect was observed for the absolute volume of fluid intake during smaller and larger volumes of fluid intake (*F*(1, 26) = 14.25, *p* = 0.001). *Post-hoc* analyses and mean values for each stage of the treatment revealed different TWI volumes for HIGH and LOW, when each was consuming a smaller volume of water (Table 1). This difference could have impacted the urinary, hematological, and thirst findings. Therefore, all analyses were corrected with the absolute value of TWI change from days 1–2 to 3–6 as a covariate.

### 3.3. Hydration Markers

Urine volume increased (*F*(1, 25) = 6.06, *p* = 0.02) and urine osmolality decreased (*F*(1, 25) = 4.35, *p* = 0.04) as TWI increased within both groups (Table 2). A significant interaction effect (TWI × group) was identified for urine volume (*F*(1, 25) = 11.94, *p* = 0.002), and urine osmolality (*F*(1, 25) = 8.76, *p* = 0.007). No significant effect of TWI or group on serum osmolality existed throughout the entire investigation: HIGH days 1–3, 293 ± 3 mOsm·kg^−1^; days 4–7, 294 ± 2 mOsm·kg^−1^, LOW days 1–3, 295 ± 4 mOsm·kg^−1^; days 4–7, 293 ± 2 mOsm·kg^−1^.

### 3.4. Hormone Response

**Figure 2:**
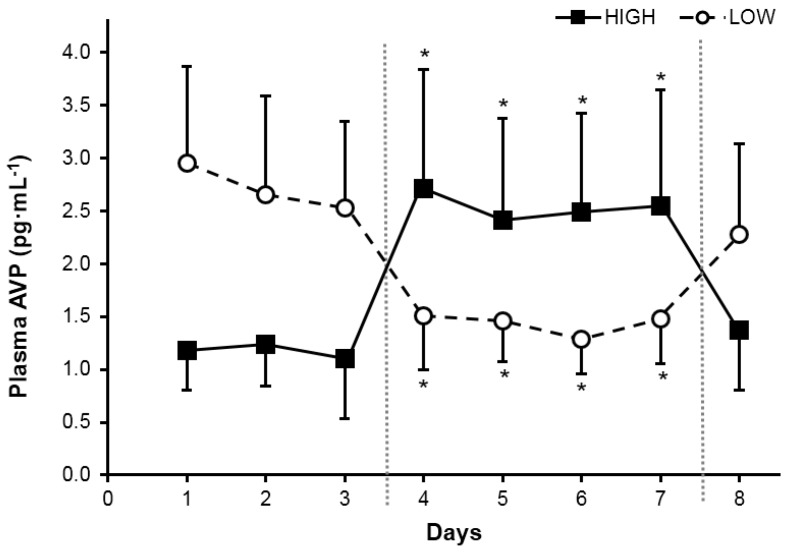
Plasma arginine vasopressin (AVP) concentrations in morning plasma samples of HIGH and LOW during *ad libitum* baseline (three days), controlled water intake (four days), and *ad libitum* recovery (one day). Different phases separated by vertical dotted lines. * Significant difference within group from the three-day mean of baseline (*p* < 0.001).

**Figure 3:**
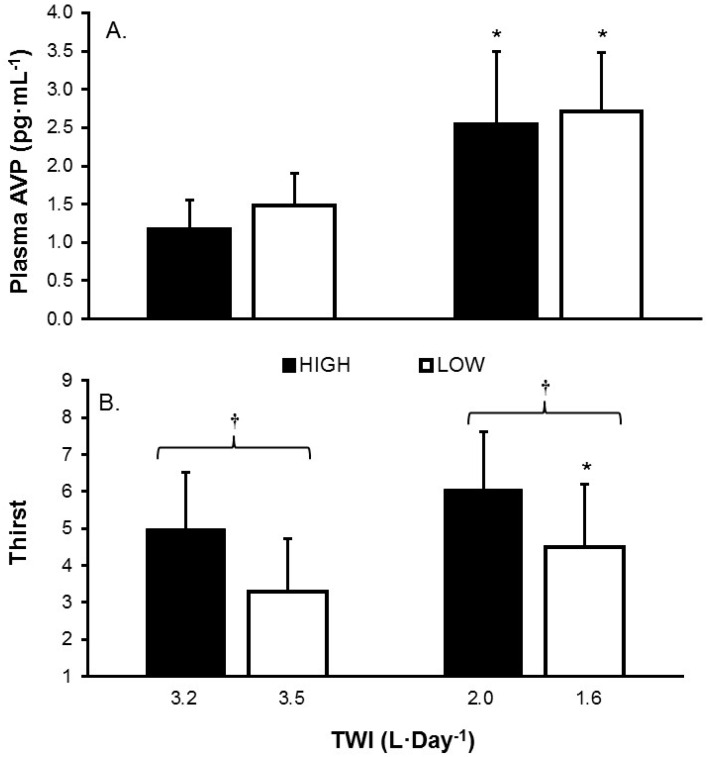
(**A**) Plasma AVP concentration; and (**B**) thirst rating, grouped on the basis of similar TWI for HIGH and LOW. * Significant within-group differences, when consuming different TWI. **^†^** represents significant between-group differences (HIGH *versus* LOW, *p* = 0.002), when consuming similar TWI.

A significant main effect of TWI on plasma AVP concentration existed (*F*(1, 24) = 98.43, *p* < 0.001). Plasma AVP also was analyzed over time and a significant effect of day (Figure 2) was observed for both HIGH (*F*(5, 60) = 21.39, *p* < 0.001) and LOW (*F*(5, 26) = 18.87, *p* < 0.001); only AVP concentrations during the treatment (days 3–6) differed from baseline time points. During recovery (*ad libitum* fluid consumption, days 7–8), AVP concentrations were similar to those observed during baseline, for both groups. No between-group differences were observed when groups were consuming similar total water volumes (Figure 3A).

The mean serum aldosterone concentration in morning serum samples exhibited no main effect of TWI or group, and no significant interaction as a result of the treatment (HIGH, days 1–2, 3.14 ± 1.70; HIGH, days 3–6, 3.17 ± 0.90; LOW, days 1–2, 3.95 ± 1.04; LOW, days 3–6, 3.32 ± 0.72 pg·mL^−1^).

### 3.5. Perception of Thirst

**Figure 4:**
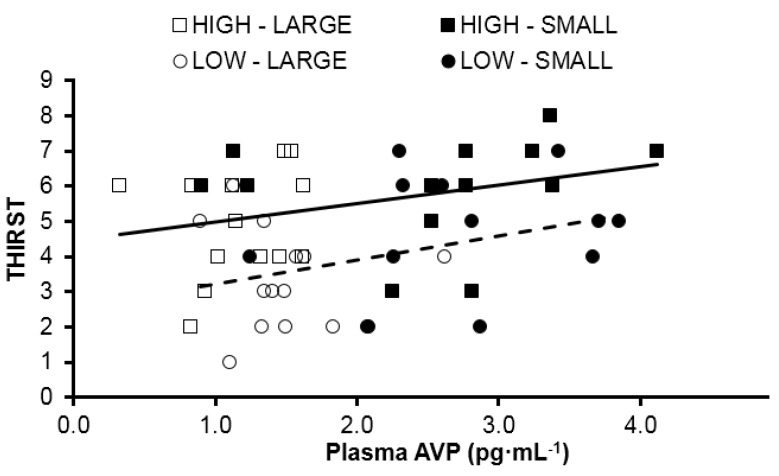
Relationship between thirst rating and plasma AVP concentration. The y-intercepts of these lines are significantly different (*p* < 0.001). Squares represent HIGH, circles represent LOW. Open markers represent when each group was consuming a SMALL volume of fluid, and filled markers represent when each group was consuming a LARGE volume of fluid. Solid line represents line of best fit for all data points from HIGH. Dashed line represents line of best fit for all data points from LOW.

The mean rating of thirst was compared when HIGH and LOW consumed similar TWI during the baseline and treatment periods (Figure 3B). A main effect of TWI (*F*(1, 13) = 18.70, *p* = 0.001) was observed. Also, a main effect of group (*F*(1, 13) = 8.03, *p* = 0.014) also was observed, with HIGH consistently rating thirst greater than LOW, regardless of TWI. The relationships between plasma AVP concentration and thirst rating appear in Figure 4 for HIGH (*F*(1, 24) = 2.98, *p* = 0.10, *R*^2^ = 0.11) and LOW (*F*(1, 24) = 3.44, *p* = 0.08, *R*^2^ = 0.13). The regression lines of best fit in this figure have equivalent slopes (*t*(48) = 0.327, *p* = 0.74), but the y-intercepts are significantly different (*t*(49) = 3.694, *p* < 0.001).

## 4. Discussion

The present investigation furthers our understanding of the complex effects of habitual high and low *ad libitum* total water consumption of women, within a range (1.6 to 3.5 L·day^−1^; Table 1) that is commensurate with the adequate intake reference values of the Institute of Medicine, National Academy of Sciences (IOM, 2.7 L·day^−1^) [5], and the European Food Safety Authority (EFSA, 2.0 L·day^−1^) [7]. Both HIGH and LOW displayed similar concentrations of AVP at large and small volumes of daily fluid intake, as well as a similar change in AVP concentrations between daily intake volumes. These changes were effective at stabilizing serum osmolality in both groups, as was expected. Added, or restricted water intake appeared to be managed by urinary loss or conservation despite an observed interaction between group and TWI. For example, when both groups were consuming a large volume of water, mean water intake for HIGH was approximately 3.2 L·day^−1^, or 0.3 L·day^−1^ less than LOW (3.5 L·day^−1^). In this instance HIGH excreted approximately 0.3 L·day^−1^ less of slightly more concentrated urine. The main difference between the groups appeared in perception of thirst. Thirst was elevated in HIGH at all times, even after accounting for the small differences in fluid intake between groups at each prescriptive level (*i.e.*, large and small). Together these findings tell us that in free-living conditions, habitual high and low drinkers both maintain fluid balance. However, their perception of thirst may be the driving force behind their habitual intake. Thus, it appears that these groups are physiologically similar in terms of their management of large and small volumes of water.

Similar to three publications by Perrier and colleagues [6,24,32], the present study utilized an experimental design comparing individuals who habitually consumed high and low volumes of fluid *ad libitum*. The present database confirms their two fundamental observations regarding the general population: (a) habitual water intake is a determinant of urinary volume and concentration *(i.e.*, not excessive water loss via sweat or urine); and (b) urine volume and osmolality are well-suited to track subtle differences in the hydration process which are modulated by TWI. The present investigation also extends the work of Perrier *et al*. in the following ways. Although AVP was determined to be a defining factor between HIGH and LOW at rest [6], the response within these two distinct groups to fluid modification was not apparent. This can now be associated with similar trends in urinary markers of hydration from our past research [25]. Secondly, in the investigation where fluid intake was altered [24] the test participants resided in France, were in-patients with tightly controlled water intakes, and were aged 25–40 years, whereas in the present study, young American women (20 ± 2 years) exhibited a range of TWI and were free-living. These are important distinctions because differences of renal and thirst sensitivity exist for men and women [33], and cultural or regional differences of dietary practices and learned drinking behaviors (*i.e.*, amount of salt consumption, inclusion of alcohol with meals) influence fluid-electrolyte balance and renal function in the general population [34].

Lastly, circulating AVP and its association with thirst due to their mutual response to osmoreceptors located within organum vasculosum of the lamina terminalis and the hypothalamus has been well established [35,36], to the extent that one can validly predict the other [37], though differences in this relationship due to habitual TWI has yet to be described. Figure 4 illustrates the relationship between AVP and thirst for both HIGH and LOW (although the relationship failed to reach significance due to our small sample size). However, a significant difference of the y-intercepts indicates that there is a different hormonal stimulus for water conservation related to increasing levels of thirst between groups. HIGH demonstrated elevated thirst throughout the investigation, even at the small volume measurement, when *post-hoc* analysis revealed that HIGH was consuming significantly more water than LOW (2.0 ± 0.2 *versus* 1.6 ± 0.4 L). It is unclear if the elevated thirst in HIGH is a “secondary” (*i.e.*, not related to body water deficit) drinking behavior that results from hedonic compensation [38], or if the elevated fluid consumption, potentially driven by individual thoughts and feelings of its benefit, could be the cause of slightly elevated blood volume and a resetting of the thirst-related central osmoreceptors [39]. Both of these explanations are supported by the mean difference in starting bodyweight and the previously mentioned difference in estimated plasma volume within this sample [25].

Regardless, it has previously been observed that the plasma osmolality threshold for AVP secretion can range as far as 276 to 291 mOsm∙kg^−1^ and the threshold for stimulation of thirst can range as far as 289–299 mOsm∙kg^−1^ across individuals [40,41]. Thus, the most logical explanation is that genetic variation in osmoreceptor threshold, which has been demonstrated in the animal model [42], may predispose an individual to their habitual daily fluid intake. At rest HIGH and LOW demonstrated similar serum osmolalities. Thus, when individuals are grouped based on their habitual water intake we may be separating those with a low thirst threshold (*i.e.*, HIGH) from those with a high thirst threshold (*i.e.*, LOW). Conversely, we may have also separated those from a low AVP threshold (*i.e.*, LOW) from those with a high AVP threshold (*i.e.*, HIGH). It is not possible given the current experimental design to confirm this. However, because both groups were confirmed to be in fluid balance, it would appear that they differ on the mechanism on which they rely most. Body water is conserved in HIGH through increased dependence on water intake and LOW maintains homeostasis due to increased water conservation.

Despite the above mentioned strengths, the methodology employed does have limitations that constrain full interpretation of the results. First, the slight difference in fluid volumes between groups at each prescriptive level was not purposeful. All efforts during the subject identification phase of the investigation were made to establish a single volume that would bring each group to the habitual TWI of the other group during the intervention. However, small differences between food, sodium, and fluid intake during observation and the experimental phases, as well as differences in water content of food may have contributed to dissimilarities between groups. Follow-up investigations should employ water and food prescription during both “habitual” water intake phases and “modified” water intake phases to limit any small discrepancy. Second, the levels of AVP observed are slightly higher than those reported in the literature, with few samples falling below the detectable limit [35]. We attribute this finding to the methodology of the concentration of samples prior to analysis as well as the sample timing taking place in the morning, prior to significant water consumption. Although the values may be troublesome when seeking for an absolute comparison, the trends identified between groups within this intervention remain clear. Finally, although the “recovery” period was added to help address the participants return to habitual TWI, neither the fluid modification period nor the recovery were long enough to establish any meaningful observations of adaptation, should they exist.

## 5. Conclusions

The following four statements summarize our findings. Firstly, the mean 24 h TWI of 120 healthy young women in the present investigation (2.3 ± 0.8 L·day^−1^) and the 24 h TWI distribution (Figure 1) support the adequate intake recommendations for water of IOM [5] (2.7 L·day^−1^) and EFSA [7] (2.0 L·day^−1^). Secondly, women at the extremes of normal daily fluid consumption (HIGH and LOW) are physiologically and perceptually distinct during their habitual drinking behavior (Table 2, Figure 4). Thirdly, the mean perceived thirst of HIGH significantly exceeded that of LOW, at a given TWI (Figure 3). Fourthly, AVP, not aldosterone, was primarily responsible for maintenance of body water and tonicity across days in both HIGH and LOW (Figure 2). Within college aged women there is a difference in perception of thirst and the subsequent drive to drink at the two tail ends of the TWI continuum. Now that it is apparent that the thirst drive between these groups are indeed different, the next step will be to determine how AVP and thirst change during progressive plasma osmolality manipulation. Such an experiment would enable us to determine if a lower AVP threshold in LOW enables water balance with less water intake, while a lower thirst threshold in HIGH enables water balance with less secretion of AVP.

## Figures and Tables

**Table 1:** Mean 24 h TWI (L·day^−1^) for women in the HIGH and LOW groups, during the experimental intervention.

	Baseline: 2 Days *Ad Libitum* (Days 1–2, 2–3) ^a^	Treatment: 4 Days Modified TWI (Days 3–4, 4–5, 5–6, 6–7) ^a^	Recovery: 1 Day Ad Libitum (Days 7–8) ^b^
HIGH (*n* = 14)	3.2 ± 0.6 *^,†^	2.0 ± 0.2 *^,†^	3.2 ± 0.9
LOW (*n* = 14)	1.6 ± 0.4 *^,†^	3.5 ± 0.1 *^,†^	1.7 ± 0.5

Abbreviation: TWI, total water intake (water + beverages + moisture in solid foods). ^a^ baseline and treatment values were used to compare group responses to different TWI volumes; ^b^ recovery values were used for regression analyses and to evaluate hormone responses across time; * baseline and treatment means were significantly different: all *p* < 0.001; ^†^ HIGH and LOW were significantly different: *p* = 0.002.

**Table 2:** Mean (±SD) urinary and serum osmolality values when HIGH and LOW consumed similar TWI.

Variable	Group	Total Water Intake	
		3.2–3.5 L·Day^−1^	1.6–2.0 L·Day^−1^
24 h Urine Volume (L·day^−1^) *^,†^	HIGH	1.9 ± 0.6	1.2 ± 0.2 ^‡,§^
	LOW	2.2 ± 0.4	0.8 ± 0.3 ^‡,§^
Urine Osmolality (mOsm·kg^−1^) *^,†^	HIGH	392 ± 129 ^§^	592 ± 222 ^‡,§^
	LOW	274 ± 60 ^§^	766 ± 210 ^‡,§^
Serum Osmolality (mOsm·kg^−1^)	HIGH	293 ± 3	294 ± 2
	LOW	293 ± 2	295 ± 4

* Main effect of TWI. ^§^ Significant difference between groups HIGH and LOW. ^†^ Significant interaction (TWI level x group). ^‡^ Significant difference between TWI levels (columns 3 and 4), within group.
